# Comparative analysis of deutocerebral neuropils in Chilopoda (Myriapoda): implications for the evolution of the arthropod olfactory system and support for the Mandibulata concept

**DOI:** 10.1186/1471-2202-13-1

**Published:** 2012-01-03

**Authors:** Andy Sombke, Elisabeth Lipke, Matthes Kenning, Carsten HG Müller, Bill S Hansson, Steffen Harzsch

**Affiliations:** 1 Ernst Moritz Arndt University of Greifswald, Zoological Institute and Museum, Cytology and Evolutionary Biology, 17487 Greifswald, Germany; 2 Max Planck Institute for Chemical Ecology, Department of Evolutionary Neuroethology, 07745 Jena, Germany; 3 Ernst Moritz Arndt University of Greifswald, Zoological Institute and Museum, General Zoology and Zoological Systematics, 17487 Greifswald, Germany; 4 RWTH Aachen University, Institute of Biology II, Unit of Developmental Biology and Morphology of Animals, 52065 Aachen, Germany

## Abstract

**Background:**

Originating from a marine ancestor, the myriapods most likely invaded land independently of the hexapods. As these two evolutionary lineages conquered land in parallel but separately, we are interested in comparing the myriapod chemosensory system to that of hexapods to gain insights into possible adaptations for olfaction in air. Our study connects to a previous analysis of the brain and behavior of the chilopod (centipede) *Scutigera coleoptrata *in which we demonstrated that these animals do respond to volatile substances and analyzed the structure of their central olfactory pathway.

**Results:**

Here, we examined the architecture of the deutocerebral brain areas (which process input from the antennae) in seven additional representatives of the Chilopoda, covering all major subtaxa, by histology, confocal laser-scan microscopy, and 3D reconstruction. We found that in all species that we studied the majority of antennal afferents target two separate neuropils, the olfactory lobe (chemosensory, composed of glomerular neuropil compartments) and the corpus lamellosum (mechanosensory). The numbers of olfactory glomeruli in the different chilopod taxa ranged from ca. 35 up to ca. 90 and the shape of the glomeruli ranged from spheroid across ovoid or drop-shape to elongate.

**Conclusion:**

A split of the afferents from the (first) pair of antennae into separate chemosensory and mechanosensory components is also typical for Crustacea and Hexapoda, but this set of characters is absent in Chelicerata. We suggest that this character set strongly supports the Mandibulata hypothesis (Myriapoda + (Crustacea + Hexapoda)) as opposed to the Myriochelata concept (Myriapoda + Chelicerata). The evolutionary implications of our findings, particularly the plasticity of glomerular shape, are discussed.

## Background

In arthropod phylogeny the emerging consensus is that Myriapoda are not to be considered the closest relatives of Hexapoda anymore (Tracheata concept), but rather that hexapods constitute a sister group or even an in-group of Crustacea (Tetraconata concept; e.g. [1-4]). Hence, it seems well established that from a marine ancestor of Euarthropoda, members of the Chelicerata as well as the Myriapoda and Hexapoda invaded land independently from each other [5,6]. The successful transition from marine to terrestrial life requires a number of physiological adaptations that are important for survival out of water. The sensory organs of terrestrial species must be able to function in air rather than in water and hence were exposed to new selection pressures that may have reshaped the nervous system (see e.g. [7-10] for examples on terrestrial Crustacea). We are interested in how the structure of the central nervous system mirrors functional adaptations of the olfactory system to a terrestrial life style. Studying the olfactory system in Myriapoda and comparing it to that of Hexapoda may provide insights into how the arthropod nervous system evolved in response to new environmental and ecological challenges.

**Figure 1:**
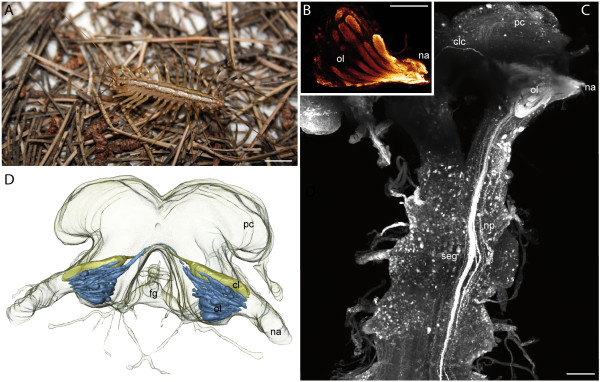
**Scutigeromorpha**. **A ***Scutigera coleoptrata*. **B **Single optical section of a neurobiotin backfill showing an olfactory lobe with distinct olfactory glomeruli. cLSM scan. **C **cLSM scan (maximal projection) of the brain and the subesophageal ganglion. View from ventral. Left antennal nerve was filled with neurobiotin. Antennal neurites project into the seg. **D **3D reconstruction of the brain of *S. coleoptrata *with deutocerebral neuropils. Blue: olfactory glomeruli, yellow: corpus lamellosum. **Abbreviations: cl **corpus lamellosum, **clc **contralateral connection, **fg **frontal ganglion, **na **nervus antennalis, **np **neurite projections, **ol **olfactory lobe, **pc **protocerebrum, **seg **subesophageal ganglion. **Scalebars: **A = 10 mm, B, C = 100 μm.

**Figure 2:**
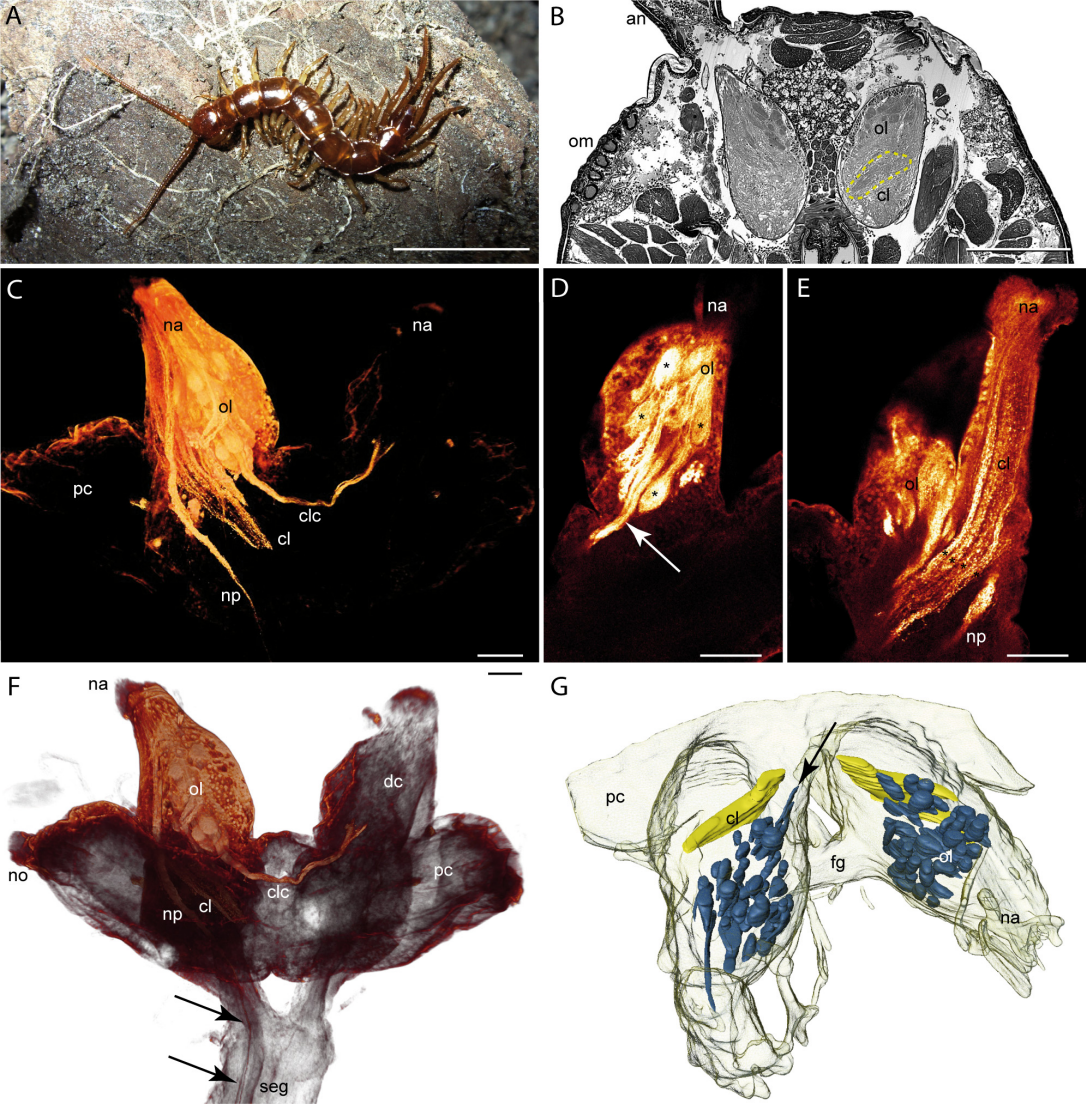
**Lithobiomorpha**. **A ***Lithobius forficatus*. **B **Histological horizontal section of the head showing the deutocerebral lobes with olfactory glomeruli and corpus lamellosum (dashed line) as well as the ommatidia. **C **Voltexrendering (Amira) of a neurobiotin backfill showing deutocerebral neuropils and neurite projections. **D **Horizontal optical section (cLSM scan) of a neurobiotin backfill (dorsal deutocerebrum) showing single olfactory glomeruli (asterisks). The arrow points to the contralateral connection of the olfactory lobe. **E **Horizontal optical section (cLSM scan) of a neurobiotin backfill (ventral deutocerebrum) showing the corpus lamellosum. Asterisks mark single lamellae of the neuropil. **F **Voltexrendering (Amira) of a neurobiotin backfill showing the brain with deutocerebral neuropils and projections. Arrows point to antennal neurites projecting into the subesophageal ganglion. **G **3D reconstruction of the brain with deutocerebral neuropils. Blue = olfactory glomeruli, yellow = corpus lamellosum. Contralateral connection of OG and CL is not shown. **Abbreviations: an **antenna, **cl **corpus lamellosum, **clc **contralateral connection, **dc **deutocerebrum, **fg **frontal ganglion, **na **nervus antennalis, **no **nervus opticus, **np **neurite projections, **ol **olfactory lobe, **om **ommatidia, **pc **protocerebrum, **seg **subesophageal ganglion. **Scalebars: **A = 10 mm, B = 500 μm, C-E = 100 μm.

**Figure 3:**
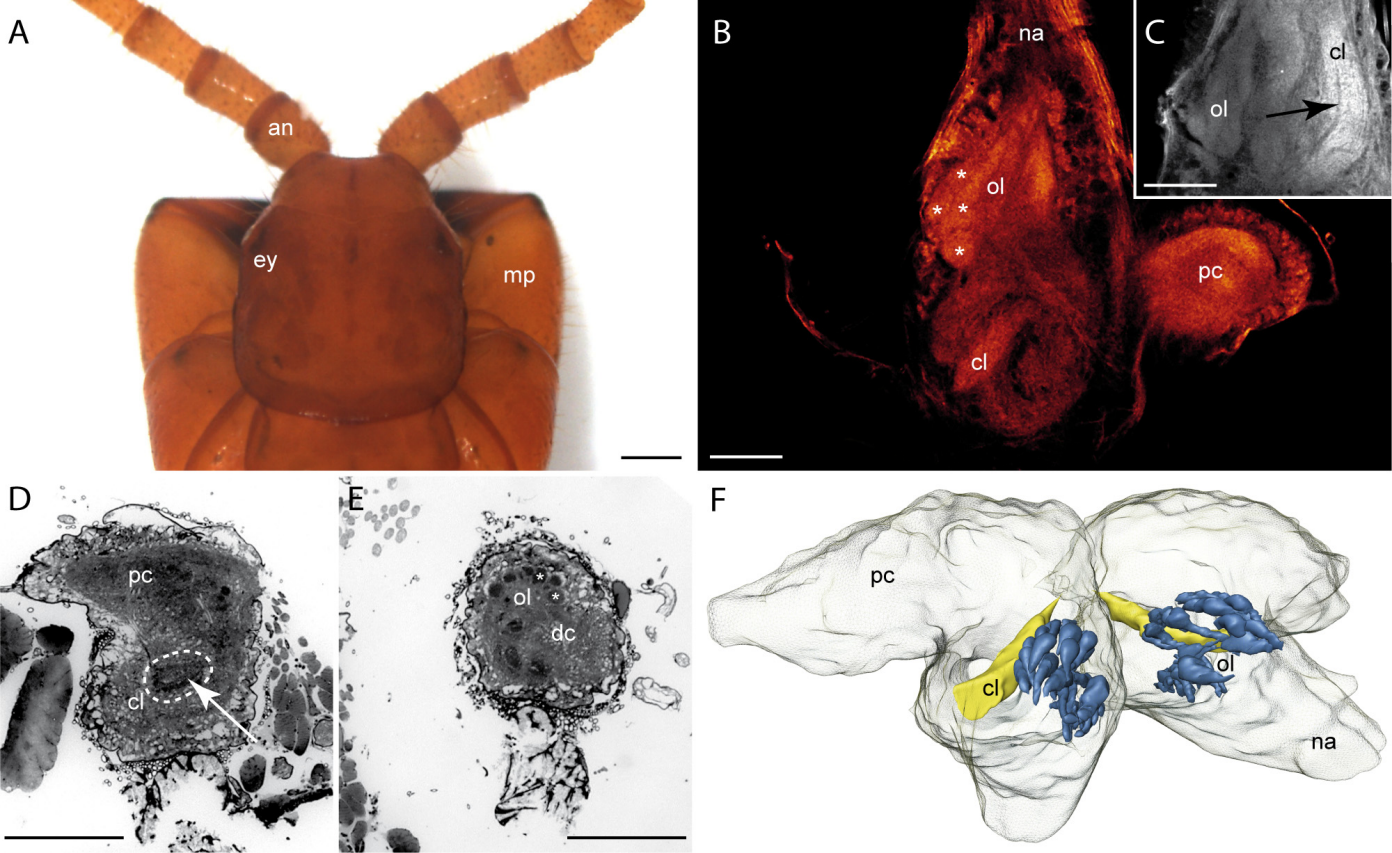
**Craterostigmomorpha**. **A **Head and maxillipedes of *Craterostigmus tasmanianus *from dorsal. **B **Horizontal optical section of an autofluorescence preparation (cLSM stack). Single olfactory glomeruli (asterisks) are weakly detectable. **C **Different horizontal section of the same preparation as in B. The arrow marks the structural composition of the corpus lamellosum. **D **Histological cross section of the right brain hemisphere showing the proto- and deutocerebrum with the corpus lamellosum (dashed line). **E **Histological cross section of the left deutocerebrum showing the dorsomedian located olfactory glomeruli. **F **3D reconstruction of the brain with deutocerebral neuropils. Blue = olfactory glomeruli, yellow = corpus lamellosum. Contralateral connection of the CL is not shown. **Abbreviations: an **antenna, **cl **corpus lamellosum, **dc **deutocerebrum, **ey **eye, **mp **maxillipede, **na **nervus antennalis, **ol **olfactory lobe, **pc **protocerebrum. **Scalebars: **A = 1 mm, B-E = 100 μm.

**Figure 4:**
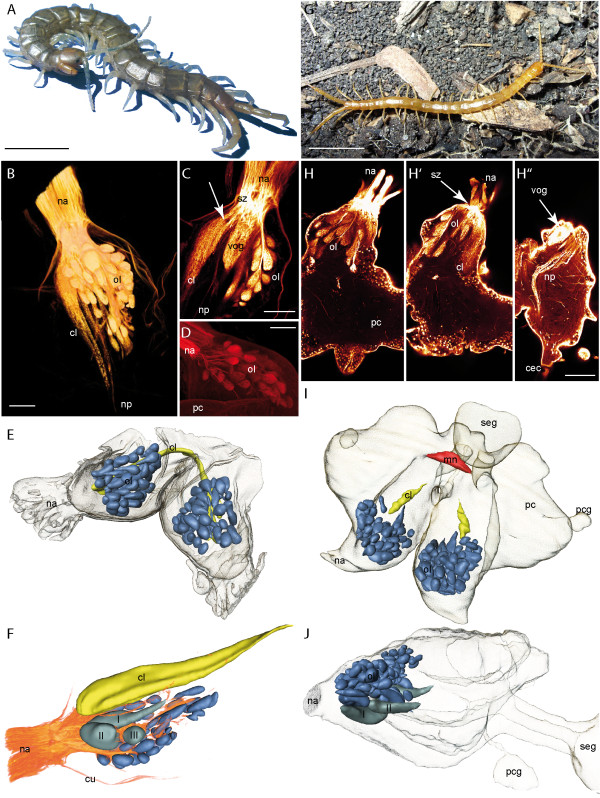
**Scolopendromorpha**. **A ***Scolopendra oraniensis*. **B **Neurobiotin backfill of the antennal nerve in *S. oraniensis *showing the olfactory lobe, the corpus lamellosum, and neurite projections (horizontal maximal projection, cLSM scan). **C **Single optical horizontal section of a Lucifer yellow backfill in *S. oraniensis *(cLSM scan). Antennal neurites cross each other in a sorting zone and project into different neuropils. The arrow marks the structural composition of the corpus lamellosum in which single lamellae are weakly noticeable. The large ventral OG is visible in this section. Single olfactory glomeruli in the olfactory lobe are arranged like in a grape. **D **Neurobiotin backfill of the antennal nerve of *S. oraniensis*. Only a subpopulation of the antennal neurites and olfactory glomeruli is labeled (horizontal maximal projection, cLSM scan). **E **3D reconstruction of the brain of *Scolopendra subspinipes *(dorsal protocerebrum is not shown) with deutocerebral neuropils. Blue = olfactory glomeruli, yellow = corpus lamellosum. **F **3D reconstruction of deutocerebral neuropils of *Scolopendra oraniensis *combined with volume rendering of the antennal backfill in B. Three enlarged ventral glomeruli (I, II, III) are present. **G ***Cryptops hortensis*. **H **Single horizontal optical sections (cLSM) of a neurobiotin backfill of the right antennal nerve in *C. hortensis *from dorsal to ventral. Antennal nerve bundles and innervation of single olfactory glomeruli. **H' **Sorting zone (arrow) of antennal neurites and corpus lamellosum. **H'' **Larger ventral olfactory glomerulus (arrow) and neurite projections. **I **3D reconstruction of the brain of *C. hortensis *with deutocerebral neuropils and midline neuropil. Contralateral connection of the CL is not shown. Blue = olfactory glomeruli, yellow = corpus lamellosum, red = midline neuropil. **J **Lateral view of the 3D reconstruction in I. Two enlarged ventral glomeruli (I, II) are present. **Abbreviations: cec **circumesophageal connectives, **cl **corpus lamellosum, **mn **midline neuropil, **na **nervus antennalis, **np **neurite projections, **ol **olfactory lobe, **pc **protocerebrum, **pcg **protocerebral gland, **seg **subesophageal ganglion, **sz **sorting zone, **vog **ventral olfactory glomerulus. **Scalebars: **A and F = 10 mm, B-D, G = 100 μm.

**Figure 5:**
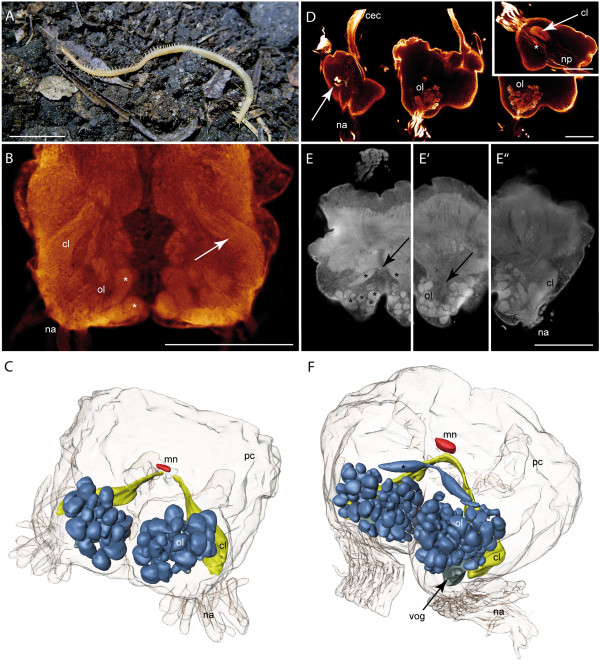
**Geophilomorpha**. **A ***Geophilus carpophagus*. **B **Single horizontal optical section (cLSM) of an autofluorescence preparation of the brain of *Haplophilus subterraneus *showing olfactory glomeruli (asterisks) and the structural composition of the corpus lamellosum (arrow). **C **3D reconstruction of the brain of *H. subterraneus *with deutocerebral neuropils and midline neuropil. Blue = olfactory glomeruli, yellow = corpus lamellosum, red = midline neuropil. Contralateral connection of the CL is not shown. **D **Single horizontal optical sections (cLSM) of a neurobiotin backfill of the right antennal nerve in *Stigmatogaster dimidiatus *from ventral to dorsal. Left: several somata stained by the neurobiotin backfill (arrow) and neurite projections into a circumesophageal connective. Middle: Antennal nerves and olfactory glomeruli. Right: The slightly concave appearance of the olfactory lobe. Inset: Sagittal optical section of the same preparation showing the structural composition of the corpus lamellosum (arrow) and neurite projections (asterisk). **E **Single horizontal optical sections (cLSM) of an autofluorescence preparation of the brain of *S. dimidiatus *from dorsal to ventral. Olfactory glomeruli (asterisks) and the contralateral connection between the posteroventral OG (arrow). **E' **Concave appearance of the olfactory lobe (arrow). **E'' **ventrolateral position of the corpus lamellosum. **F **3D reconstruction of the brain of *S. dimidiatus *with deutocerebral neuropils and midline neuropil. Blue = olfactory glomeruli, gray = bigger ventral olfactory glomerulus, yellow = corpus lamellosum, red = midline neuropil. **Abbreviations: cec **circumesophageal connective, **cl **corpus lamellosum, **mn **midline neuropil, **na **nervus antennalis, **np **neurite projections, **ol **olfactory lobe, **pc **protocerebrum, **vog **ventral olfactory glomerulus. **Scalebars: **A = 5 mm, B, D, E = 100 μm.

The Chilopoda together with the Progoneata (Symphyla + (Diplopoda + Pauropoda)) constitute the taxon Myriapoda. The position of monophyletic Myriapoda within the Euarthropoda is still under debate and most of the recent phylogenetic studies either place them as sister group to the Tetraconata (Crustacea + Hexapoda) together forming the taxon Mandibulata (e.g. [11,12]) or as a sister group to the Chelicerata to form the taxon Myriochelata (e.g. [13]). The Chilopoda are one of the few arthropod taxa of which the internal phylogeny appears to be widely accepted [14]. The Notostigmophora (Scutigeromorpha) (Figure 1A) are the sister group to the Pleurostigmophora which are composed of Lithobiomorpha (Figure 2A) and Phylactometria. In the latter taxon, the Craterostigmomorpha (Figure 3A) are the sistergroup to the Epimorpha which are composed of Scolopendromorpha (Figure 4A, G) and Geophilomorpha (Figure 5A) [14].

Our knowledge of the chilopod nervous system largely relies on studies from the 19^th ^and early 20^th ^century using paraffin sections and light microscopy (e.g. [15-20]). Studies with contemporary neuroanatomical methods are only available for the brain, and specifically for the deutocerebrum (the second brain neuromere) of *Scutigera coleoptrata *[21].

The deutocerebrum in the mandibulate (Myriapoda + (Crustacea + Hexapoda)) brain is associated with the first pair of antennae and is characterized by a unified architecture: it comprises a paired anterior olfactory lobe that receives the chemosensory afferents from the first antennae, and (at least) a paired posterior neuropil [21,22]. These uni- or bipartite posterior neuropils are thought to process mechanosensory stimuli and have a range of different names within the mandibulate taxa: antennal mechanosensory and motor center (AMMC) or dorsal lobe in Hexapoda (e.g. [23]), corpus lamellosum in Chilopoda [19-21,24] and lateral antennular neuropil (LAN) plus median antennular neuropil (MAN) in malacostracan Crustacea and Remipedia [8,25-29]. All of these structures can be unified under the term mechanosensory neuropils.

The chemosensory olfactory lobe (called antennal lobe in Hexapoda) is composed of structural and functional subunits [22], which are called olfactory glomeruli (or olfactory neuropils) [21,24]. These subunits are clearly demarcated dense neuropils in which the axons of olfactory sensory neurons (OSN) terminate and interact with olfactory interneurons *via *the first synapses of the olfactory pathway [22,30]. Thus, within the olfactory glomeruli of Hexapoda, malacostracan Crustacea and the House Centipede *Scutigera coleoptrata*, first order integration of olfactory input takes place, which is then relayed to secondary brain centers *via *olfactory projection neurons (e.g. [8,21,22]). The glomerular array in hexapods is thought to represent a chemotopic map, which forms the basis of the olfactory code [31-33]. Based on this uniform architecture and several additional synapomorphic characters [22], the olfactory system in general as well as the olfactory glomeruli in particular were suggested to represent homologous structures within the deutocerebrum of the Mandibulata [21,22], whereas previously, also a convergent evolution was proposed [3].

Nevertheless, previous studies have revealed a high degree of plasticity in the shape and arrangement of mandibulate olfactory glomeruli, suggesting a critical evaluation of glomerular neuropils. In *Scutigera coleoptrata*, the olfactory glomeruli are elongated and arranged in parallel [21]. On the contrary, in many decapod Crustacea the olfactory lobes consist of glomeruli that are cone-like and in the lobe are arranged with their apices pointing inwards (reviews: [22,27,28,34]). In some decapods crustaceans, these glomeruli may be extremely elongated [8,10], whereas studies on representatives of the basal malacostracan taxon *Nebalia *(Leptostraca) suggest spherical glomeruli to be part of the malacostracan ground pattern (Kenning and Harzsch; unpublished results). Such spherical glomeruli are also present in marine Isopoda [9]. Furthermore, it has been well documented from crayfish (Astacidea), spiny lobsters (Palinuroidea) and hermit crabs (Paguroidea) that in the olfactory lobe each glomerulus is stratified and provides an outer cap, a subcap, and a base [8,10,34]. Most pterygote insects also feature spherical glomeruli [22], whereas primarily flightless hexapods diverge from this pattern [35,36].

Is the shape of olfactory glomeruli of purely functional significance or does it contain an unexplored phylogenetic signal? Clearly, comparative information on the deutocerebral neuropils in a broad range of myriapods will contribute to this question. This study sets out to analyze the architecture of the central olfactory pathway in Chilopoda in more detail. To that end we analyzed the brains of representatives of eight chilopod species using histology, confocal laser scanning microscopy (cLSM), and 3D reconstruction. Our data are compared and evaluated with regard to the evolution of glomerular shape in Mandibulata.

## Results

### General morphology of the chilopod brain

**Figure 6:**
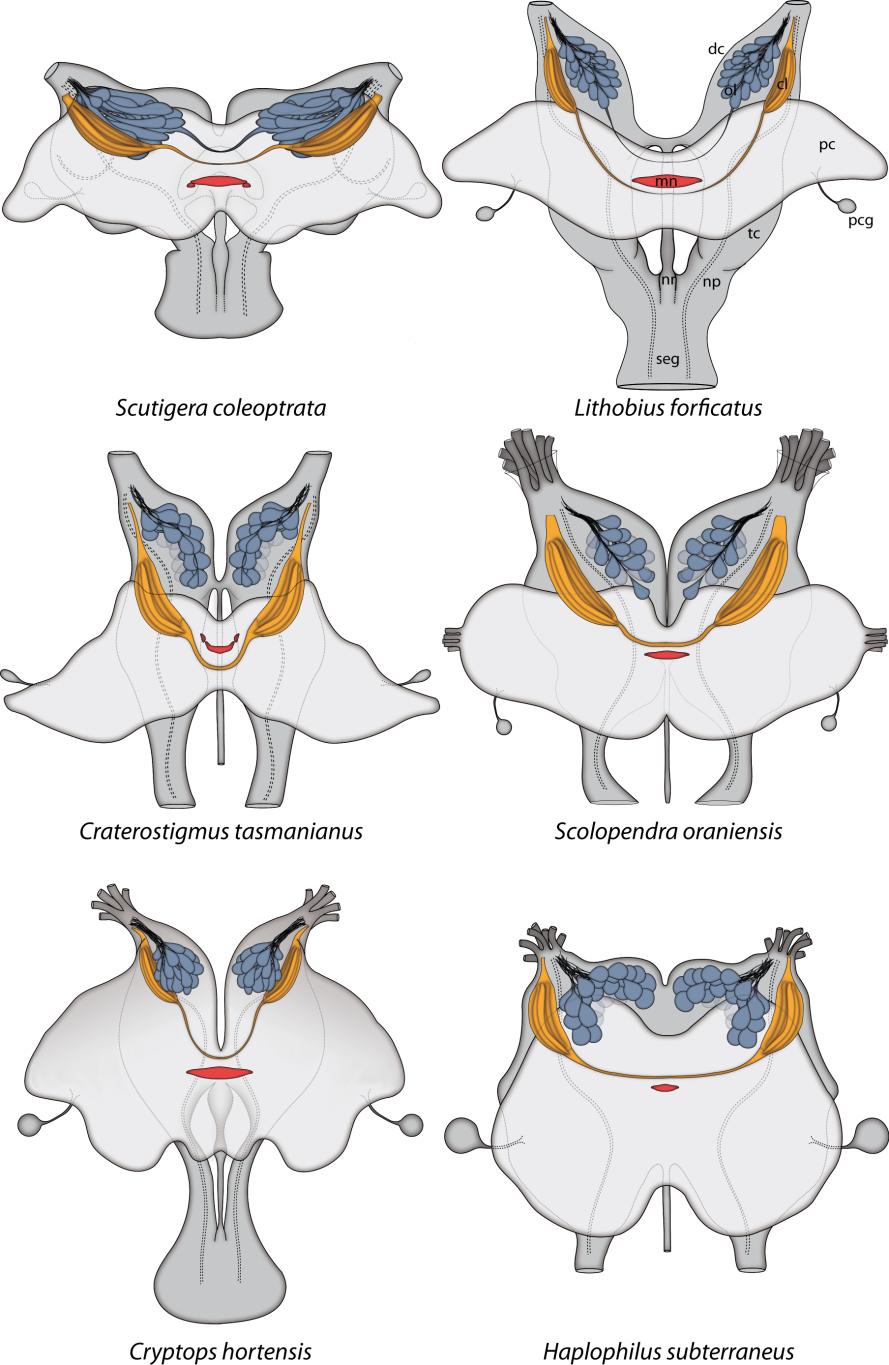
**Brains of selected Chilopoda**. Schematic representation of the brains of selected Chilopoda with illustration of the olfactory glomeruli (blue), corpus lamellosum (yellow), Midline neuropil (red) and neurite projections (dashed lines). View from dorsal. The protocerebrum appears brighter. **Abbreviations: cl **corpus lamellosum, **dc **deutocerebrum, **mn **midline neuropil, **np **neurite projection, **nr **nervus recurrens, **ol **olfactory lobe, **pc **protocerebrum, **pcg **protocerebral gland, **seg **subesophageal ganglion, **tc **tritocerebrum.

Due to the anteriorly projecting antennae in the Chilopoda, the deutocerebrum (DC) is the most anterior part of the brain with regard to the body axis, so that the protocerebrum is always located dorsally and extends into lateral lobes where the optic neuropils are located (Figure 1, 2, 3, 4, 5). In the blind Cryptopidae (Scolopendromorpha) (Figure 4G), these lateral lobes are much smaller and are even totally reduced in the Geophilomorpha (Figure 5C, F). Contrary to Fahlander [20], who stated that a distinct midline neuropil is lacking in Geophilomorpha, all investigated representatives exhibit an unpaired midline neuropil (Figure 6). In *S. coleoptrata *and *C. tasmanianus *this unpaired neuropil is associated with small lateral lobes. As this study focuses on the organization of deutocerebral neuropils, we will not further consider here, if these neuropils represent an equivalent of the crustacean and hexapod central bodies or the chelicerate arcuate bodies (compare [3,37]).

The morphology of the sensory antennal nerve differs in investigated chilopod species: while Scutigeromorpha, Lithobiomorpha and Craterostigmomorpha exhibit a "solitary" and robust antennal nerve, in Scolopendromorpha and Geophilomorpha it is composed of a bundle of several discrete nerves (Figure 4E, H; 5C, D, F; 6). Both types of antennal nerves enter the DC at its frontolateral or frontal edges (compare review [38]). The motoric antennal nerve will not further considered here. In the following the sensory antennal nerve is referred as antennal nerve.

Apart from the Geophilomorpha, the anterior part of the deutocerebrum is separated into two discrete hemispheres (Figure 1D, 2F, 3F, 4E, H; 5C, F). Anterograde backfilling experiments reveal that the antennal nerve targets the deutocerebral neuropils, which therefore are first order processing areas in the brain. In all Chilopoda examined, each deutocerebral hemisphere contains an olfactory lobe (OL) being composed of densely packed olfactory glomeruli (OG) and a corpus lamellosum (CL). A central coarse neuropil in the OL is not present in most examined taxa (uncertain for the Geophilomorpha). In the following, bilaterally paired structures will be referred in singular. A demarcation between deutocerebrum and tritocerebrum is not clearly apparent, although the stomodeal bridge and frontal connectives indicate the anterior margin of the tritocerebrum (compare [21,24,38])

### Scutigeromorpha

The organization of deutocerebral neuropils in *Scutigera coleoptrata *(Figure 1A) was described in detail by Sombke et al. [21] and therefore will be only briefly reviewed here. Due to the roundish head outline, the shape of the brain differs from that of the pleurostigmophoran chilopod taxa. The antennal nerve enters the brain at its frontolateral edge (Figure 1D) and divides into two branches: an anterior part innervates the olfactory glomeruli whereas the posterior part innervates the corpus lamellosum. In *S. coleoptrata*, the olfactory lobes are arranged in an angle of nearly 180° to each other (Figure 1D, 6). Histological sections and dextran-biotin backfills reveals that single olfactory glomeruli have an elongated shape and are arranged in a parallel array (Figure 1B, D; and [21]). A 3D reconstruction (Figure 1D) reveals the bilateral symmetrical pattern with two contralaterally connected glomeruli (anterior deutocerebral commissure *sensu *Fahlander [20]) (Figure 1C, D: clc). In all histological section series and autofluorescence preparations, a total number of 34 distinct and uniquely identifiable OG in a more or less invariant arrangement is present (Figure 1D) [21]. The posterior part of the antennal nerve innervates the presumed mechanosensory neuropil called corpus lamellosum (CL; [19,21,24]), which in *S. coleoptrata *is composed of approximately eight parallel neuropilar lamellae [21]. Two different types of lamellae were recognized: the outer lamellae forming a distal connection, and inner lamellae that extend further dorsomedially to project towards the contralateral hemisphere (posterior deutocerebral commissure *sensu *Fahlander [20,21]). Golgi impregnations shows that axons targeting the CL are much thicker than those targeting the OG and give off short side branches alongside their length [21]. In backfills of the antennal nerve, we found that the dye was also transported along thicker neurites projecting into the ventrolateral protocerebrum and the subesophageal ganglion (Figure 1C) [21].

### Lithobiomorpha

The lithobiomorph head is flattened, a fact that is mirrored in the shape of the brain (reviewed in [24]). The antennal nerve enters the deutocerebrum at its frontal edge (Figure 2B-G). The deutocerebrum is organized in an anterior olfactory lobe (OL) with glomeruli (OG) and a posterior corpus lamellosum (CL) (Figure 2B-G). In contrast to *S. coleoptrata*, the OLs extend in a slightly dorsomedian direction resulting in an angle of nearly 90° (Figure 2G, 6). Antennal afferents were revealed by neurobiotin backfills which, in addition to the terminations in the OL and CL, also show a bundle of neurites projecting from the antennal nerve through the tritocerebrum deep into the subesophageal ganglion (Figure 2C, F: np). Within the OL, two OG feature a contralateral connection (Figure 2C, F: clc, D: arrow). Histological sections and neurobiotin backfills reveal that single OG have a drop-like to elongated shape that narrows to their anterodistal edges (Figure 2D, G). All OG appear compact without any subcompartments. The 3D reconstruction reveals a bilaterally symmetrical pattern with a total number of 43 OG (Figure 2G). The CL is located posteriorly to the OG and extends a small contralateral connection (not shown). The neuropil is composed of at least four lamellae (Figure 2C, E asterisks). However, the lamellae are more densely packed than in *S. coleoptrata *so that a precise count is not possible.

### Craterostigmomorpha

This is the first investigation of the nervous system of *Craterostigmus tasmanianus *(Figure 3A). Like in the Lithobiomorpha, the head of the Craterostigmomorpha is flattened, which is reflected in the shape of the brain (Figure 3F). The robust antennal nerve enters the deutocerebrum at its frontal edge (Figure 3B, F). In principle, the deutocerebrum is organized in an anteromedian OL and a posterior CL (Figure 3B-F). The OLs extend in a median direction resulting in an angle of nearly 90° (Figure 3F, 6). Histological sections and autofluorescence preparations reveal that single OG have a drop-like to elongated shape with a nearly circular profile and a smaller diameter distally (Figure 3B, E, F). The OG are arranged in an anteroposterior direction (Figure 3B, F). A contralateral connection of the OLs was not found. The 3D reconstruction shows a total number of 36 OG (Figure 3F). The CL is located posteriorly to the OL and features a thin contralateral connection (not illustrated). Due to the fixation, a lamellar organization was not clearly recognizable. However, a partition into discrete lamellae is likely (Figure 3C, D arrow).

### Scolopendromorpha

Similar to the Lithobiomorpha and Craterostigmomorpha, the head and also the brain of the Scolopendromorpha are flattened. The DC is innervated by several antennal nerve bundles (Figure 4E, H) at its frontal edge. The DC is composed of an anteriorly located olfactory lobe and a posteriorly located CL (Figure 4B-E, H, I). The OLs extend in a slightly dorsomedian direction resulting in an angle of less than 90° (Figure 4B, E, H, I). The OG are arranged in an anteroposterior direction. In the three investigated scolopendromorph species, the shape of the OG appears spheroid to drop-like elongated (Figure 4B-F, H-J). In *Scolopendra oraniensis *and *Cryptops hortensis*, ventral glomeruli are much bigger (Figure 4C, F, H, J). In *S. oraniensis*, three enlarged ventral OG are present (Figure 4F) while in *C. hortensis *two enlarged ventral OG exist (Figure 4J). A contralateral connection of the OLs is absent. Based on histological sections, backfill experiments, and autofluorescence preparations, all OG appear compact without any subcompartments and are arranged in a bilaterally symmetrical pattern. Numbers of OG range from 51 in *Scolopendra subspinipes *(Figure 4E), across 56 in *Cryptops hortensis *(Figure 4H) to 58 in *Scolopendra oraniensis *(Figure 4F). The CL is located posteroventrally to the OL. A contralateral connection of the CL is always present, although it varies in thickness in the three investigates species. In *Scolopendra subspinipes*, it appears as a thick connection (Figure 4E) while in *Cryptops hortensis *it appears very thin (not shown in Figure 4I). Single lamellae are not clearly detectable. However, backfill experiments reveal an alternating texture within the neuropil (Figure 4B, C arrow, G). Neurobiotin backfills reveal an additional neurite bundle projecting from the antennal nerve through the tritocerebrum into the subesophageal ganglion (Figure 4B, C, H: np).

### Geophilomorpha

The brain of obligatory blind Geophilomorpha is spherical in shape and also the most modified within the Chilopoda [24,38]. The dominant component of the geophilomorph brain is the deutocerebrum. A clear demarcation between proto- and deutocerebrum is not detectable (Figure 5C, F). The antennal nerve comprises 10-15 bundles of sensory neurons and innervates the DC at its frontal edge (Figure 5B-F). The deutocerebral hemispheres are fused posteromedially (Figure 5B-F). The OLs are arranged more or less parallel to each other (Figure 5B, C, F). In both investigated species, the OL is composed of spheroid to slightly ovoid OG. In *Stigmatogaster dimidiatus*, the OL appears slightly invaginated posteriorly (Figure 5D, E: arrow) thus resulting in a cup-like shape. The number of OG is 49 in *Haplophilus subterraneus *and 97 in *Stigmatogaster dimidiatus*. A conspicuous contralateral connection (clc) features the OLs of *S. dimidiatus*, where two elongated OG extend a thin clc (Figure 5E: arrow, F: asterisks). However, in *H. subterraneus*, a clc of the OLs is not present. The OG appear compact without any subcompartments and a bilateral symmetry seems to be present (Figure 5B-F). In both investigated species, an enlarged ventral glomerulus is found (Figure 5F: vog; not shown in the reconstruction of *H. subterraneus*). The CL is located posteroventrally to the OL (Figure 5B-F) and features a thin contralateral connection (Figure 5F). In the autofluorescence preparations, this thin connection could not be depicted clearly. In most of the preparations, the CL appears lamellar (Figure 5B, D inset; arrows). Posteriorly directed antennal neurite projections were revealed by neurobiotin backfills, which showed a bundle of neurites projecting from the antennal nerve, through the tritocerebrum (asterisk in inset Figure 5D) and probably into the subesophageal ganglion *via *the circumesophageal connectives (Figure 5D: cec). Interestingly, several somata were filled by neurobiotin in the ventral brain (Figure 5D left: arrow) branching intensively in an anteroposterior direction. Whether these somata belong to projection- or local interneurons remains unknown.

## Discussion

### Chilopoda: the antennal nerve innervates two separate deutocerebral neuropils

Supporting the descriptions of Seifert [38] and Fahlander [20], we found that a sensory antennal nerve composed of several discrete bundles (as opposed to one "solitary" nerve) occurs only in representatives of the Scolopendromorpha and Geophilomorpha. In a phylogenetic view, this feature can be regarded as an apomorphy for the taxon Epimorpha.

**Figure 7:**
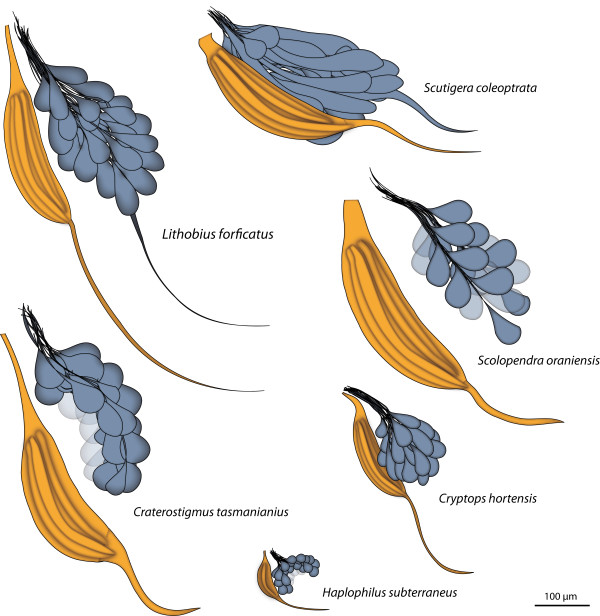
**Deutocerebral neuropils of selected Chilopoda**. Schematic representation of the deutocerebral neuropils in representatives of the Chilopoda (left hemisphere). Horizontal view with equal scaling.

Fahlander [20] described the nervous system of various Chilopoda and also interpreted the results from Saint-Rémy [15] and Hörberg [19] in a broad comparative study. Although previous authors mentioned a glomerular organized antennal lobe for the Chilopoda (e.g. [17-20,39]), the number, organization, and structural composition remained unclear. Sombke et al. [21] reinvestigated the deutocerebral neuropils in *Scutigera coleoptrata *using a variety of histological and immunhistochemical methods. Similar to *S. coleoptrata *[21], the deutocerebrum of the Chilopoda investigated here is organized into structured neuropils that can be divided into two different regions: olfactory lobe and corpus lamellosum (Figure 6, 7). Moreover, a similar organization of deutocerebral neuropils may be present in representatives of the Diplopoda ([40], Seefluth and Sombke unpublished data.) as well as in representatives of the Hexapoda and Crustacea [e.g. [23,27]].

### The chilopod olfactory lobes and olfactory glomeruli

In principle, the olfactory lobe (OL) extends from the entrance of the antennal nerve into the brain on towards the dorsomedian brain and is composed of olfactory glomeruli (OG) which are located in the anterior part of the deutocerebrum. The OG are innervated from the periphery. As a result of different innervation angles of the antennal nerves, the overall orientation of the olfactory lobes differs in the Chilopoda (Figure 6). In contrast to the remaining chilopod taxa, the OL of the Geophilomorpha appears globular and slightly invaginated (Figure 6, 7). The alignment of the OG also differs in investigated taxa: while in *S. coleoptrata *the OG form more or less parallel layers, the drop-like shape in *L. forficatus, C. tasmanianus *and the representatives of the Scolopendromorpha results in a more compact arrangement.

The presence of a central coarse neuropil in the OL of Geophilomorpha is uncertain. In many hexapod taxa, the glomeruli surround a coarse neuropil e.g. Dictyoptera [41], Hymenoptera [42], Lepidoptera and Diptera (reviewed in [22]). Contrary, in Archaeognatha the OL is composed of elongated OG without a central neuropil [36]. In malacostracan Crustacea, the OG are arranged in a peripheral radial array that surrounds a loose core of neuronal processes (reviewed in [22,34]). Single glomeruli in these animals are also innervated from the periphery (reviews: [22,27,28,34]).

In *Scutigera coleoptrata*, 34 individually identifiable OG per olfactory lobe were detected repeatedly in several specimens and these glomeruli form a fixed array, so that individual OG are identifiable [21]. In the present study, glomerular numbers were only determined in few specimens, so that the numbers have to be viewed with caution. Nevertheless, we speculate that the determined numbers are taxon-specific within the Chilopoda. In *Lithobius forficatus*, 43 OG were detected, and 36 in *Craterostigmus tasmanianus*. In the investigated Scolopendromorpha, the number of OG ranges around 50-60, while in the Geophilomorpha, some variation was encountered (49 in *H. subterraneus*, 97 in *S. dimidiatus*). In hexapods, the number of olfactory glomeruli ranges from about 20 in Collembola to approx. 250 in ants (reviewed in [22,35]) and seems to be invariant within species (e.g. [42-52]). In Crustacea the number of OG varies from approximately 60 to 1300 (reviewed in [10,22,53]) but it is uncertain if crustaceans have a fixed set of OGs [53,54].

The number of glomeruli is generally thought to provide a good indication regarding how many different olfactory receptor proteins (OR) are expressed in the antenna. One OSN typically expresses a single OR, and all OSNs expressing a specific receptor project their axons to the same glomerulus. Odor input thus paints a map of activation over the glomerular array.

The size of OG is more or less taxon-specific and constant within the investigated chilopods (Figure 7). The only exceptions are the ventrally located enlarged OG in *Scolopendra oraniensis, Cryptops hortensis*, and *Stigmatogaster dimidiatus*. This is also true for the posteriormost OGs in *Scutigera coleoptrata *(compare [21]). In general, glomeruli of increased fitness-related importance tend to increase in size. Sex-specific enlargement ("macroglomeruli") are known from various hexapods e.g. moths [55], cockroaches [56] or honeybees [42]. Other enlargements have been found to be associated with trail pheromones and with specific food cues [57]. In this study a functional correlation was not conducted, and no conclusions regarding the functional significance of macroglomeruli in Chilopoda can be drawn.

In histological sections, backfills, and autofluorescence preparations, there is not any evidence of further compartmentalization of the OG in the investigated Chilopoda as it is known from hexapods and malacostracan crustaceans. In honeybees (Hexapoda), olfactory glomeruli have a concentric organization [42,58-62], where only the periphery is innervated by axons of sensory neurons. A longitudinal subdivision of the OG into cap, subcap, and base has been well documented in crayfish, clawed and clawless lobsters, and hermit crabs (Crustacea) [8,10,63-67].

The shape of the OG in the investigated chilopods displays a considerable plasticity (Figure 6, 7). OG in the Scutigeromorpha have an elongated shape and in some the distal end is thickened and/or bent posteriorly [21]. According to Fahlander [20], the internal organization of deutocerebral neuropils in *Lithobius forficatus *strongly resembles those of the Scutigeromorpha, but here we show that the shape of the OG actually differs. In *Lithobius forficatus *and *Craterostigmus tasmanianus*, the overall shape of OG ranges from elongated (more than two times longer than wide) to drop-shaped, with a smaller anterior diameter. In the Scolopendromorpha, the shape of OG is mostly drop-like to spheroid and in the Geophilomorpha, the OG have a spheroid shape. For the Chilopoda, it is unclear if elongated or spheroid glomeruli represent the ancestral shape of the OG in this group. Here, we take benefit from the fact that the debate on the internal phylogeny of Chilopoda rather unequivocally gravitated into accepting the Pleurostigmophora concept of Verhoeff [68] in the past four decades so that we can map our results on a stable phylogeny of Chilopoda. Based on the number and position of stigmata, this phylogenetic concept separates the Scutigeromorpha (= Notostigmophora) as sister group to all other Chilopoda (Pleurostigmophora). This phylogenetic concept has received substantial support from the analysis of morphological, molecular and combined morphological-molecular data sets (e.g. [14,69-73]). If we accept this phylogeny, we have to assume that elongated OG mark the plesiomorphic state in Chilopoda, perhaps retained from the myriapod ground pattern. In this view, the exclusive occurrence of spheroid OG has to be considered an additional apomorphy of the Geophilomorpha. However, alternatives to this view are possible. In most pterygote Hexapoda, the antennal lobe (or olfactory lobe) is organized into numerous roughly spheroid OG. In Archaeognatha the OG have an elongated shape [36]. In Crustacea, the shape of olfactory glomeruli differs considerably (reviewed in [22]). The olfactory lobes of malacostracan Crustacea are typically composed of glomeruli which are columnar or wedge-shape, (reviews [3,34]). or slightly cone-shaped [9,74]. Nevertheless, certain Stomatopoda possess olfactory lobes with spheroid OG [75] as do Remipedia [29,76], Leptostraca [18], and marine isopods [9]. Within several taxa of the Chelicerata, spheroid neuropil units have been reported but these are not associated with the second brain neuromere [77-82]. Along these lines it would appear that spheroid OG within the deutocerebrum characterize both, the ground patterns of Malacostraca and Hexapoda. If this hypothesis holds true, it is parsimonious to assume that spheroid glomeruli are also part of the ground pattern of Mandibulata and would thus characterize the ground pattern of Myriapoda. In this view, the elongate shape of OG in Scutigeromorpha would be a derived character characteristic for this particular group.

In this context, the question arises if the shape of olfactory glomeruli is of purely functional significance or if it does contain an unexplored phylogenetic signal. Clearly, considering glomerular shape alone is not sufficient to answer these questions. Concerning the central olfactory pathway of malacostracan crustaceans and hexapods, the fact that in both groups the afferents of chemosensory receptor neurons terminate in lobed deutocerebral neuropils where they target neuropil units to make synaptic contacts to local olfactory interneurons and olfactory projection neurons has been suggested as evidence that the olfactory system in these two taxa goes back to a shared ground pattern [22]. Furthermore, in both taxa the axons of olfactory projection neurons link the olfactory neuropils to secondary olfactory processing centers in the protocerebrum. What is more, local olfactory interneurons in both taxa include a characteristic innervation by one or very few serotonergic giant neurons that target every OG. Hence it seem legitimate to suggest that in the ground pattern of the common ancestor of hexapods and malacostracan crustaceans, a basal computational circuit was present that included the antennal afferents, local olfactory interneurons, and projection neurons (compare [22]). Taken together, this mosaic of architectural differences as well as similarities suggests that most likely the olfactory centers and their connections are homologous in hexapods and malacostracan crustaceans, having evolved in divergent directions from a much simpler ground pattern. What we do not know at the moment is to what level of detail the connection pattern of antennal afferents with olfactory local interneurons and projection neurons in Myriapoda resembles that of Tetraconata.

### The corpus lamellosum

In a brief description of deutocerebral neuropils in *Lithobius variegatus*, Strausfeld et al. [39] described that the antennal nerve innervates the olfactory lobe and that a lateral strand projects to a region behind it, which the authors called dorsal lobe in analogy to the hexapod mechanosensory neuropil. This posterior deutocerebral neuropil in Chilopoda had already been termed "*masse lamelleuse" *by Saint-Rémy [16] and latinized by Fahlander [20] who called it corpus lamellosum (CL). Because it reflects the characteristics of this structured neuropil, we suggest maintaining this nomination. As mentioned above, in *Scutigera coleoptrata *the posterior partition of the antennal nerve innervates the CL in which approximately eight parallel lamellae were found [21]. Golgi impregnations showed that sensory neurites innervating the CL appear much thicker than those innervating the olfactory glomeruli and give off short side branches along their length [21]. Although similar Golgi experiments on other chilopod taxa have not been conducted yet, it appears to us that the architecture of the CL in the other chilopod taxa investigated here is similar to that of *S. coleoptrata*. In *S. coleoptrata*, the parallel lamellae project dorsomedially and extend into the posterior deutocerebral commissure [20,21]. By backfilling the antennal nerve in *Lithobius forficatus*, at least 4 single lamellae are visible. The report of Fahlander [20] that in *Lithobius forficatus *the CL is not composed of distinct lamellae can therefore be rejected. In *Craterostigmus tasmanianus*, a lamellation is only partially visible. In the Scolopendromorpha, backfill experiments also show an arrangement of parallel fibers suggesting a lamellation in the investigated genera. In the Geophilomorpha, the CL also appears lamellar. Possibly, due to a higher degree of condensation, single lamellae could not be detected. In summary, all the investigated chilopods exhibit a CL, which is composed of parallel lamellae and features a contralateral connection.

In pterygote Hexapoda, the first and second antennomeres of the antenna supply the dorsal lobe (mechanosensory neuropil) whereas the flagellar sensilla are mostly specialized for olfactory perception and their neurites project into the olfactory lobe [56]. Examples where mechanosensory and gustatory afferents project into the such a mechanosensory neuropil in the posterior region of the deutocerebrum (and in some cases even proceed into the anterior subesophageal ganglion) are e.g. *Periplaneta americana *[62,83], *Apis mellifera *[84], *Gryllus bimaculatus *[85,86], and *Aedes aegypti *[33,87]. In these organisms, presumptive tactile antennal afferents provide two pairs of long branches whereas several short branches are orientated laterally and form a multilayered arrangement medially in the dorsal lobe. This arrangement exhibits a similarity to the branching pattern of sensory axons in the CL of *Scutigera coleoptrata *[21]. In malacostracan crustaceans, the first (deutocerebral) pair of antennae, in addition to the aesthetasc chemosensory pathway, provides mechanosensory and non-aesthetasc chemosensory input to the lateral and median antennular neuropil (LAN and MAN) [25-28,34]. Between the lobes of the LAN, contralateral connections occur in Decapoda. The general organization of the LAN and MAN in many respects matches the innervation and connections of the CL. To summarize, we suggest that in the ground pattern of Chilopoda, Hexapoda, and Crustacea, the posterior deutocerebrum is characterized by at least one neuropil (corpus lamellosum, dorsal lobe, lateral antennular neuropil) that processes mechanosensory input from the first pair of antennae. Such a neuropil is absent in Chelicerata and therefore represents a homology of Mandibulata (apomorphic character state). However, the architecture of this neuropil was then elaborated in different ways in the various mandibulate lineages.

### Posterior neurite projections

In all investigated Chilopoda (except for *C. tasmanianus*), antennal afferents also project into the subesophageal ganglion and even into the ventral nerve cord. These neurites project ipsilaterally and bypass the deutocerebral neuropils. In addition, in *S. coleoptrata *a second projection to the ventrolateral protocerebrum was found [21]. We speculate that these posterior neurites may project to a gustatory or motoric center in the subesophageal ganglion. In Hexapoda, certain neurites from the antennal nerve also project to the subesophageal ganglion and the thoracic ganglia [62,88-90]. Barrozo et al. [90] suggested that these neurite projections with a characteristic larger diameter might serve to insure a rapid neuronal transmission of sensory inputs towards centers responsible for controlling motor activities and physiological processes. In Crustacea, these characteristic posterior neurite projections have not yet been described. As a consequence it can be assumed, that they are reduced within the Crustacea. The presence in Chilopoda and Hexapoda could indicate an additional shared feature of the mandibulate deutocerebrum. However, neurite projections are also described from the pectines in Scorpiones [81]. If these neurite projections correspond to those in Chilopoda and Hexapoda remains uncertain.

### The deutocerebrum and olfactory lobes of Euarthropoda in an evolutionary context: support for the Mandibulata concept

In the Chilopoda, the deutocerebrum is characterized by two distinct neuropil regions, which are innervated by antennal sensory afferents. The olfactory glomeruli are bilaterally and symmetrically arranged and appear presumably in a taxon-specific fixed number. Contralateral connections occur in some species. The corpus lamellosum is a structured neuropil and exhibits a contralateral connection. In the Chilopoda, antennal neurite projections transit the deutocerebrum and project into the subesophageal ganglion.

In Chilopoda, Crustacea, and Hexapoda, distinct neuropils for processing sensory information of the (first) antennae are located in the deutocerebrum. According to *Hox*-gene expression patterns and morphological investigations, the deutocerebrum and the deutocerebral antennae are homologous within Mandibulata and correspond to the chelicere neuromere in Chelicerata [91-93]. Although in some Chelicerata glomerular chemosensory processing areas associated with a sensory appendage are located in the trunk ganglia (e.g. [81]), distinct neuropils for processing of chemo- and mechanosensory information have not yet been reported for their second brain neuromere. Moreover, a characteristic divergence of sensory neurites and the presence of a mechanosensory neuropil are not realized in Chelicerata. There is a consensus now that the antenna in Onychophora is a protocerebral appendage and therefore not equivalent to the deutocerebral antenna in Mandibulata [94-97]. In Onychophora, chemosensory centers composed of glomerular neuropils are located within the protocerebrum [98,99]. Similar to the chelicerates, separate mechanosensory neuropils associated with the antennal input do not seem to be present in onychophorans. Strausfeld and co-workers [98,99] emphasize the structural similarities of onychophoran and chelicerate brains so that we suggest that these two taxa represent the plesiomorphic arthropod character state concerning brain architecture. In summary, within the arthropod outgroups of Mandibulata, chemosensory appendages and olfactory glomeruli, if present, are never located in the second brain neuromere (deutocerebrum).

## Conclusion

Our most important conclusion is that the presence of a bifunctional deutocerebrum composed of distinct neuropils for chemo- and mechanosensory qualities is homologous in Chilopoda, Diplopoda, Hexapoda and Crustacea and can therefore be postulated as an apomorphic character complex for the Mandibulata. However, our neuroanatomical data strongly contradict a sister group relationship of Myriapoda and Chelicerata ("Myriochelata; [13]), but instead support the Mandibulata concept (e.g. [11,12]). In this view, the absence of olfactory lobes in various Crustacea (Branchiopoda and certain "Maxillopoda"; [22,100,101]) and Hexapoda (Odonata, certain Hemiptera and Coleoptera, reviewed in [22]) as well as the absence of the mechanosensory neuropils in Cephalocarida (Crustacea; [102]) can be interpreted as a reduction.

## Methods

### Experimental animals

Specimens were collected on the Balearic Island Ibiza (Spain) mainly in pine forests or in Germany mainly in litter and soil. Specimens of *Craterostigmus tasmanianus *were collected by Robert Mesibov in Tasmania. If not fixed directly after capture, individuals were kept in plastic tubes (50 ml; Carl Roth, Germany) at room temperature. For keeping of animals, they were transferred into plastic boxes supplied with bark and water. They were fed with *Drosophila melanogaster *or juveniles of *Achaeta domestica*.

Representatives of all five chilopod subtaxa were investigated: (1) *Scutigera coleoptrata *(Linnaeus, 1758), Scutigeromorpha: Scutigeridae; collected in Spain: Ibiza. (2) *Lithobius forficatus *(Linnaeus, 1758), Lithobiomorpha: Lithobiidae; collected in Germany: Aachen, Greifswald. (3) *Craterostigmus tasmanianus *Pocock, 1902, Craterostigmomorpha; collected in Australia: Tasmania. (4) *Cryptops hortensis *(Donovan, 1810), Scolopendromorpha: Cryptopidae; collected in Germany: Aachen, Greifswald. (5) *Scolopendra oraniensis *Lucas, 1846, Scolopendromorpha: Scolopendridae; collected in Spain: Ibiza. (6) *Scolopendra subspinipes *Leach, 1815, Scolopendromorpha: Scolopendridae; ordered from btbe Insektenzucht GmbH, Germany http://www.futtertiere24.de/. (7) *Haplophilus subterraneus *(Shaw, 1794), Geophilomorpha: Himantariidae; collected in Germany: Aachen, Greifswald. (8) *Stigmatogaster dimidiatus *(Meinert, 1870), Geophilomorpha: Himantariidae; collected in Spain: Ibiza.

### Histology

For section series, several individuals were anesthetized, decapitated and prefixed for 24 h in a solution of 80% ethanol, 37% formaldehyde and 100% acetic acid (10:4:1). After washing in sodium hydrogen phosphate buffer (PBS, pH 7.4), specimens were postfixed for 1 h in 2% OsO_4 _solution (same buffer) at room temperature and, following dehydration in a graded series of acetone, embedded in Araldite (Araldite epoxy resin kit, Agar Scientific). Serial semithin sections (1-1.5 μm) were prepared with a Microm HM 355 S rotary microtome and stained using 1% toluidine blue and Pyronin G in a solution of 1% sodium tetraborate.

### Autofluorescence preparation

For autofluorescence analysis, specimens were anesthetized and decapitated. Dissected brains were fixed in a solution of 4% paraformaldehyde and 4% glutaraldehyde (1:1) for at least one week at 4°C. After several washing steps in PBS, brains were dehydrated in a graded series of ethanol and embedded in methyl salycilate. For cLSM, an excitation of 488 nm was used to detect autofluorescence from the nervous tissue.

### Antennal Backfilling

For antennal backfills, specimens were anesthetized and mounted in plastic Petri-dishes. One antenna was cut and the antennal nerve was exposed. For neurobiotin backfills, the antennal nerve stump was isolated in petroleum jelly, covered by aqua dest. for two minutes, and subsequently exposed to 5% neurobiotin (Vector Laboratories) being dissolved in aqua dest. Preparations were incubated at 4°C for 1 day. After final dissection and fixation in 4% paraformaldehyde for 24 hours, the preparations were washed in several changes of PBS and incubated in streptavidin conjugated to Cy3 (1:2000, Jackson Immunoresearch) for 24 hours. After washing in several changes of PBS, the preparations were dehydrated in a graded series of ethanol and mounted in methyl salycilate. In controls the brains of which were not subjected to backfills, incubation in streptavidin alone resulted in an absence of all labeling. For Lucifer yellow backfills, the treatment of the antennal nerve stump was the same as for neurobiotin backfills, but instead of washing and incubating, the preparations were directly dehydrated in a graded series of ethanol after fixation and mounted in methyl salycilate.

### Microscopy, 3D reconstruction, and terminology

Wholemounts and brain sections were examined with a Nikon eclipse 90i microscope and a Leica SP 5 II confocal laser scanning microscope (cLSM). All images were processed with Adobe Photoshop using global contrast and brightness adjustment features.

The alignment and 3D reconstruction was made using AMIRA 5.1 (Visage Imaging) operated on a FS Celsius work station. In each section, contours of the nervous system and neuropilar regions were demarcated and a 3D reconstruction was generated. The 3D reconstruction of the brain of *Craterostigmus tasmanianus *was generated by merging two reconstructions of single brain hemispheres of the same specimen.

The neuroanatomical terminology is according to [30].

## Authors' contributions

AS and EL conducted the sampling, preparation and fixation of brains, backfill experiments, and the 3D reconstructions. MK prepared the schematic representations of the chilopod brains. AS drafted the main part of the manuscript and all other authors assisted in drafting the manuscript. All authors read and approved the final manuscript.

## References

[B1] Harzsch S Neurophylogeny: architecture of the nervous system and a fresh view on arthropod phylogeny International Journal of Biological Sciences 2006 46 162 194 10.1093/icb/icj011 21672733

[B2] Harzsch S The architecture of the nervous system provides important characters for phylogenetic reconstructions: examples from the Arthropoda Species, Phylogeny and Evolution 2007 1 33 57

[B3] Strausfeld NJ Brain organization and the origin of insects: an assessment Proceedings of the Royal Society B: Biological Sciences 2009 276 1929 1937 10.1098/rspb.2008.1471 19324805 PMC2677239

[B4] Strausfeld NJ Andrew DR A new view of insect-crustacean relationships I. Inferences from neural cladistics and comparative neuroanatomy Arthropod Structure and Development 2011 40 276 288 10.1016/j.asd.2011.02.002 21333750

[B5] Grimaldi D Engel MS Evolution of the Insects 2005 Cambridge: Cambridge University Press

[B6] Rota-Stabelli O Telford MJ A multi criterion approach for the selection of optimal outgroups in phylogeny: Recovering some support for Mandibulata over Myriochelata using mitogenomics Molecular Phylogenetics and Evolution 2008 48 103 111 10.1016/j.ympev.2008.03.033 18501642

[B7] Hansson BS Harzsch S Knaden M Stensmyr M Breithaupt T and Thiel M The neural and behavioral basis of chemical communication in terrestrial crustaceans Chemical Communication in Crustaceans 2010 New York: Springer 149 173

[B8] Harzsch S Hansson BS Brain architecture in the terrestrial hermit crab *Coenobita clypeatus *(Anomura, Coenobitidae), a crustacean with a good aerial sense of smell BMC Neuroscience 2008 9 58 10.1186/1471-2202-9-58 18590553 PMC2459186

[B9] Harzsch S Rieger V Krieger J Seefluth F Strausfeld NJ Hansson BS Transition from marine to terrestrial ecologies: Changes in olfactory and tritocerebral neuropils in land-living isopods Arthropod Structure & Development 2011 40 244 257 10.1016/j.asd.2011.03.002 21641866

[B10] Krieger J Sandeman RE Sandeman DC Hansson BS Harzsch S Brain architecture of the largest living land arthropod, the Giant Robber Crab *Birgus latro *(Crustacea, Anomura, Coenobitidae): evidence for a prominent central olfactory pathway? Frontiers in Zoology 2010 7 25 10.1186/1742-9994-7-25 20831795 PMC2945339

[B11] Regier JC Shultz JW Zwick A Hussey A Ball B Wetzer R Martin JW Cunningham CW Arthropod relationships revealed by phylogenomic analysis of nuclear protein-coding sequences Nature 2010 463 1079 1083 10.1038/nature08742 20147900

[B12] Rota-Stabelli O Campbell L Brinkmann H Edgecombe GD Longhorn SJ Peterson KJ Pisani D Philippe H Telford MJ A congruent solution to arthropod phylogeny: phylogenomics, microRNAs and morphology support monophyletic Mandibulata Proceedings of the Royal Society B: Biological Sciences 2011 278 298 306 10.1098/rspb.2010.0590 20702459 PMC3013382

[B13] Pisani D Poling L Lyons-Weiler M Hedges S The colonization of land by animals: molecular phylogeny and divergence times among arthropods BMC Biology 2004 2 1 10.1186/1741-7007-2-1 14731304 PMC333434

[B14] Edgecombe GD Giribet G Evolutionary biology of centipedes (Myriapoda: Chilopoda) Annual Review of Entomology 2007 52 151 170 10.1146/annurev.ento.52.110405.091326 16872257

[B15] Saint-Rémy G Sur la structure du cerveau chez les Myriapodes et les Arachnides Revue biologique du Nord de la France 1889 8 281 298

[B16] Saint-Rémy G Contribution a l'étude du cerveau chez les arthropods trachéates Arcives de zoologie experimentale et generale 1887 2 1 274

[B17] Holmgren NF Zur vergleichenden Anatomie des Gehirns: Von Polychaeten, Onychophoren, Xiphosuren, Arachniden, Crustaceen, Myriapoden und Insekten. Vorstudien zu einer Phylogenie der Arthropoden Kungliga Svenska Vetenskapsakademiens Handligar 1916 56 1 315

[B18] Hanström B Vergleichende Anatomie des Nervensystems der wirbellosen Tiere: Unter Berücksichtigung seiner Funktion 1928 Berlin: Springer

[B19] Hörberg T Studien über den komparativen Bau des Gehirns von *Scutigera coleoptrata *L Lunds Universitets Årsskrift N.F. Avd. 2 1931 27 1 2421179864

[B20] Fahlander K Beiträge zur Anatomie und systematischen Einteilung der Chilopoden Zoologiska Bidrag från Uppsala 1938 17 1 148

[B21] Sombke A Harzsch S Hansson BS Organization of Deutocerebral Neuropils and Olfactory Behavior in the Centipede *Scutigera coleoptrata *(Linnaeus, 1758) (Myriapoda: Chilopoda) Chemical Senses 2011 36 43 61 10.1093/chemse/bjq096 20962283

[B22] Schachtner J Schmidt M Homberg U Organization and evolutionary trends of primary olfactory brain centers in Tetraconata (Crustacea + Hexapoda) Arthropod Structure & Development 2005 34 257 299 10.1016/j.asd.2005.04.00322214827

[B23] Homberg U Christensen TA Hildebrand JG Structure and function of the deutocerebrum in insects Annual Review of Entomology 1989 34 477 501 10.1146/annurev.en.34.010189.002401 2648971

[B24] Sombke A Rosenberg J Hilken G Minelli A Chilopoda-The Nervous System Treatise on Zoology-Anatomy, Taxonomy, Biology. The Myriapoda 2011 1 Leiden: Brill 217 234

[B25] Schmidt M Ache BW Antennular projections to the midbrain of the spiny lobster. II. Sensory innervation of the olfactory lobe The Journal of Comparative Neurology 1992 318 291 303 10.1002/cne.903180306 1583164

[B26] Schmidt M Ache BW Processing of antennular input in the brain of the spiny lobster, *Panulirus argus*. I. Non-olfactory chemosensory and mechanosensory pathway of the lateral and median antennular neuropils Journal of Comparative Physiology A 1996 178 579 604 10.1007/BF00227374

[B27] Sandeman DC Sandeman RE Derby CD Schmidt M Morphology of the Brain of Crayfish, Crabs, and Spiny Lobsters: A Common Nomenclature for Homologous Structures Biological Bulletin 1992 183 304 326 10.2307/1542217 29300672

[B28] Sandeman DC Scholtz G Sandeman RE Brain Evolution in Decapod Crustacea Journal of Experimental Zoology 1993 265 112 133 10.1002/jez.1402650204

[B29] Fanenbruck M Harzsch S A brain atlas of *Godzilliognomus frondosus *Yager, 1989 (Remipedia, Godzilliidae) and comparison with the brain of *Speleonectes tulumensis *Yager, 1987 (Remipedia, Speleonectidae): implications for arthropod relationships Arthropod Structure & Development 2005 34 343 378 10.1016/j.asd.2005.01.00722214827

[B30] Richter S Loesel R Purschke G Schmidt-Rhaesa A Scholtz G Stach T Vogt L Wanninger A Brenneis G Döring C Faller S Fritsch M Grobe P Heuer CM Kaul S Möller OS Müller CHG Rieger V Rothe BH Stegner MEJ Harzsch S Invertebrate neurophylogeny: suggested terms and definitions for a neuroanatomical glossary Frontiers in Zoology 2010 7 29 10.1186/1742-9994-7-29 21062451 PMC2996375

[B31] Galizia CG Menzel R Odour perception in honeybees: coding information in glomerular patterns Current Opinion in Neurobiology 2000 10 504 510 10.1016/S0959-4388(00)00109-4 10981621

[B32] Galizia CG Menzel R The role of glomeruli in the neural representation of odours: results from optical recording studies Journal of Insect Physiology 2001 47 115 130 10.1016/S0022-1910(00)00106-2 11064019

[B33] Ignell R Hansson BS Projection patterns of gustatory neurons in the suboesophageal ganglion and tritocerebrum of mosquitoes The Journal of Comparative Neurology 2005 492 214 233 10.1002/cne.20691 16196031

[B34] Schmidt M Mellon D Breithaupt T, Thiel M Neuronal Processing of Chemical Information in Crustaceans Chemical Communication in Crustaceans 2010 New York: Springer 123 147

[B35] Kollmann M Huetteroth W Schachtner J Brain organization in Collembola (springtails) Arthropod Structure & Development 2011 40 304 316 10.1016/j.asd.2011.02.003 21420507

[B36] Mißbach C Harzsch S Hansson BS New insights into an ancient insect nose: The olfactory pathway of *Lepismachilis y-signata *(Archaeognatha: Machilidae) Arthropod Structure & Development 2011 40 317 333 10.1016/j.asd.2011.03.004 21665539

[B37] Strausfeld NJ Crustacean-insect relationships: the use of brain characters to derive phylogeny amongst segmented invertebrates Brain, Behavior and Evolution 1998 52 186 206 10.1159/000006563 9787219

[B38] Seifert G Das stomatogastrische Nervensystem der Chilopoden Zoologische Jahrbücher Abteilung für Anatomie und Ontogenie der Tiere 1967 84 167 190

[B39] Strausfeld NJ Buschbeck EK Gomez RS Breidbach O and Kutsch W The arthropod mushroom body: its functional roles, evolutionary enigmas and mistaken identities The Nervous Systems of Invertebrates: An Evolutionary and Comparative Approach 1995 Basel: Birghäusler Verlag 349 382

[B40] Nguyen Duy-Jacquemin M Arnold G Spatial organization of the antennal lobe in *Cylindroiulus punctatus *(Leach) (Myriapoda: Diplopoda) International Journal of Insect Morphology and Embryology 1991 20 205 214 10.1016/0020-7322(91)90010-7

[B41] Boeckh J Tolbert LP Synaptic organization and development of the antennal lobe in insects Microscopy Research and Technique 1993 24 260 280 10.1002/jemt.1070240305 8431606

[B42] Galizia CG McIlwrath SL Menzel R A digital three-dimensional atlas of the honeybee antennal lobe based on optical sections acquired by confocal microscopy Cell and Tissue Research 1999 295 383 394 10.1007/s004410051245 10022959

[B43] Chambille I Rospars JP Deutocerebron de la blatte *Blaberus craniifer *Burm. (Dictyoptera: Blaberidae): etude qualitative et identification morphologique des glomerules International Journal of Insect Morphology and Embryology 1981 10 141 165 10.1016/S0020-7322(81)80019-0

[B44] Rospars JP Invariance and sex-specific variations of the glomerular organization in the antennal lobes of a moth, *Mamestra brassicae*, and a butterfly, *Pieris brassicae* The Journal of Comparative Neurology 1983 220 80 96 10.1002/cne.902200108 6643719

[B45] Rospars JP Hildebrand JG Anatomical identification of glomeruli in the antennal lobes of the male sphinx moth *Manduca sexta* Cell and Tissue Research 1992 270 205 227 10.1007/BF00328007 1451169

[B46] Laissue PP Reiter CH Hiesinger PR Halter S Fischbach KF Stocker RF Three-dimensional reconstruction of the antennal lobe in *Drosophila melanogaster* The Journal of Comparative Neurology 1999 405 543 552 10.1002/(SICI)1096-9861(19990322)405:4<543::AID-CNE7>3.0.CO;2-A 10098944

[B47] Berg BG Galizia CG Brandt R Mustaparta H Digital atlases of the antennal lobe in two species of tobacco budworm moths, the oriental *Helicoverpa assulta *(male) and the American *Heliothis virescens *(male and female) The Journal of Comparative Neurology 2002 446 123 134 10.1002/cne.10180 11932931

[B48] Huetteroth W Schachtner J Standard three-dimensional glomeruli of the *Manduca sexta *antennal lobe: a tool to study both developmental and adult neuronal plasticity Cell and Tissue Research 2005 319 513 524 10.1007/s00441-004-1016-1 15672266

[B49] Kirschner S Kleineidam CJ Zube C Rybak J Grünewald B Rössler W Dual olfactory pathway in the honeybee, *Apis mellifera* The Journal of Comparative Neurology 2006 499 933 952 10.1002/cne.21158 17072827

[B50] Ghaninia M Hansson BS Ignell R The antennal lobe of the African malaria mosquito, *Anopheles gambiae *- innervation and three-dimensional reconstruction Arthropod Structure & Development 2007 36 23 39 10.1016/j.asd.2006.06.004 18089085

[B51] Zube C Kleineidam CJ Kirschner S Neef J Rössler W Organization of the olfactory pathway and odor processing in the antennal lobe of the ant *Camponotus floridanus* The Journal of Comparative Neurology 2008 506 425 441 10.1002/cne.21548 18041786

[B52] Dreyer D Vitt H Dippel S Goetz B el Jundi B Kollmann M Huetteroth W Schachtner J 3D standard brain of the red flour beetle *Tribolium castaneum*: a tool to study metamorphic development and adult plasticity Frontiers in Systems Neuroscience 2010 4 3 20339482 10.3389/neuro.06.003.2010 PMC2845059

[B53] Beltz BS Kordas K Lee MM Long JB Benton JL Sandeman DC Ecological, evolutionary, and functional correlates of sensilla number and glomerular density in the olfactory system of decapod crustaceans The Journal of Comparative Neurology 2003 455 260 269 10.1002/cne.10474 12454990

[B54] Blaustein DN Derby CD Simmons RB Beall AC Structure of the brain and medulla terminalis of the spiny lobster *Panulirus argus *and the crayfish *Procambarus clarkii*, with an emphasis on olfactory centers Journal of Crustacean Biology 1988 8 493 519 10.2307/1548686

[B55] Bretschneider F Über die Gehirne des Eichenspinners und des Seidenspinners (*Lasiocampa quercus *L. und *Bombyx mori *L.) Jenaische Zeitschriften für Naturwissenschaften 1924 60 563 578

[B56] Rospars JP Structure and development of the insect antennodeutocerebral system International Journal of Insect Morphology and Embryology 1988 17 243 294 10.1016/0020-7322(88)90041-4

[B57] Dekker T Ibba I Siju KP Stensmyr MC Hansson BS Olfactory shifts parallel superspecialism for toxic fruit in *Drosophila melanogaster *sibling, *D. sechellia* Current Biology 2006 16 101 109 10.1016/j.cub.2005.11.075 16401429

[B58] Pareto A Die zentrale Verteilung der Fühlerafferenz bei Arbeiterinnen der Honigbiene, *Apis mellifera *L Cell and Tissue Research 1972 131 109 140 5073638

[B59] Arnold G Masson C Budharugsa S Comparative study of the antennal lobes and their afferent pathway in the worker bee and the drone (*Apis mellifera*) Cell and Tissue Research 1985 242 593 605

[B60] Fonta C Sun XJ Masson C Morphology and spatial distribution of bee antennal lobe interneurones responsive to odours Chemical Senses 1993 18 101 119 10.1093/chemse/18.2.101

[B61] Sun XJ Fonta C Masson C Odour quality processing by bee antennal lobe interneurones Chemical Senses 1993 18 355 377 10.1093/chemse/18.4.355

[B62] Nishino H Nishikawa M Yokohari F Mizunami M Dual, multilayered somatosensory maps formed by antennal tactile and contact chemosensory afferents in an insect brain The Journal of Comparative Neurology 2005 493 291 308 10.1002/cne.20757 16255033

[B63] Sandeman DC Luff SE The structural organization of glomerular neuropile in the olfactory and accessory lobes of an Australian freshwater crayfish, *Cherax destructor* Cell and Tissue Research 1973 142 37 61 4356034 10.1007/BF00306703

[B64] Sandeman DC Sandeman RE Electrical responses and synaptic connections of giant serotonin-immunoreactive neurons in crayfish olfactory and accessory lobes The Journal of Comparative Neurology 1994 341 130 144 10.1002/cne.903410111 8006219

[B65] Langworthy K Helluy S Benton J Beltz B Amines and peptides in the brain of the American lobster: immunocytochemical localization patterns and implications for brain function Cell and Tissue Research 1997 288 191 206 10.1007/s004410050806 9042786

[B66] Schmidt M Ache BW Immunocytochemical analysis of glomerular regionalization and neuronal diversity in the olfactory deutocerebrum of the spiny lobster Cell and Tissue Research 1997 287 541 563 10.1007/s004410050778 9027299

[B67] Wachowiak M Diebel C Ache B Local interneurons define functionally distinct regions within lobster olfactory glomeruli Journal of Experimental Biology 1997 200 989 1001 9318790 10.1242/jeb.200.6.989

[B68] Verhoeff KW Bronn HG Klasse Chilopoda 1902 5 II Leipzig: Akademische Verlagsgesellschaft

[B69] Hilken G Comparison of tracheal systems and implications on phylogenetic origins Verhandlungen des Naturwissenschaftlichen Vereins Hamburg (NF) 1998 37 5 94

[B70] Wirkner CS Pass G The circulatory system in Chilopoda: functional morphology and phylogenetic aspects Acta Zoologica 2002 83 193 202 10.1046/j.1463-6395.2002.00112.x

[B71] Edgecombe GD Giribet G Adding mitochondrial sequence data (16S rRNA and cytochrome c oxidase subunit I) to the phylogeny of centipedes (Myriapoda: Chilopoda): an analysis of morphology and four molecular loci Journal of Zoological Systematics and Evolutionary Research 2004 42 89 134 10.1111/j.1439-0469.2004.00245.x

[B72] Müller CHG Vergleichend-ultrastrukturelle Untersuchungen an Augen ausgewählter Hundertfüsser (Mandibulata: Chilopoda) und zur Bedeutung von Augenmerkmalen für die phylogenetische Rekonstruktion der Euarthropoda 2008 Göttingen: Cuvillier Verlag

[B73] Shear WA Edgecombe GD The geological record and phylogeny of the Myriapoda Arthropod Structure & Development 2010 39 174 190 10.1016/j.asd.2009.11.002 19944188

[B74] Johansson KUI Hallberg E The organization of the olfactory lobes in Euphausiacea and Mysidacea (Crustacea, Malacostraca) Zoomorphology 1992 112 81 89 10.1007/BF01673809

[B75] Derby C Fortier J Harrison P Cate H Peripheral and central antennular pathway of the Caribbean stomatopod crustacean *Neogonodactylus oerstedii* Arthropod Structure & Development 2003 32 175 188 10.1016/S1467-8039(03)00048-3 18089003

[B76] Fanenbruck M Harzsch S Wägele W The brain of the Remipedia (Crustacea) and an alternative hypothesis on their phylogenetic relationships Proceedings of the National Academy of Sciences 2004 101 3868 3873 10.1073/pnas.0306212101 PMC374336 15004272

[B77] Brownell PH Glomerular Cytoarchitectures in Chemosensory Systems of Arachnids Annals of the New York Academy of Sciences 1998 855 502 507 10.1111/j.1749-6632.1998.tb10614.x 10049228

[B78] Szlendak E Oliver JH Jr Anatomy of synganglia, including their neurosecretory regions, in unfed, virgin female *Ixodes scapularis *Say (Acari: Ixodidae) Journal of Morphology 1992 213 349 364 10.1002/jmor.1052130308 1404406

[B79] van Wijk M Wadman WJ Sabelis MW Gross morphology of the central nervous system of a phytoseiid mite Experimental and Applied Acarology 2006 40 205 216 10.1007/s10493-006-9039-9 17242982

[B80] van Wijk M Wadman WJ Sabelis MW Morphology of the olfactory system in the predatory mite *Phytoseiulus persimilis* Experimental and Applied Acarology 2006 40 217 229 10.1007/s10493-006-9038-x 17245560

[B81] Wolf H The pectine organs of the scorpion, *Vaejovis spinigerus*: Structure and (glomerular) central projections Arthropod Structure & Development 2008 37 67 80 10.1016/j.asd.2007.05.003 18089128

[B82] Strausfeld N Reisenman CE Dimorphic olfactory lobes in the arthropoda Annals of the New York Academy of Sciences 2009 1170 487 496 10.1111/j.1749-6632.2009.04020.x 19686183 PMC2801554

[B83] Burdohan JA Comer CM Cellular organization of an antennal mechanosensory pathway in the cockroach, *Periplaneta americana* The Journal of Neuroscience 1996 16 5830 5843 8795635 10.1523/JNEUROSCI.16-18-05830.1996 PMC6578969

[B84] Kloppenburg P Anatomy of the antennal motoneurons in the brain of the honeybee (*Apis mellifera*) The Journal of Comparative Neurology 1995 363 333 343 10.1002/cne.903630213 8642079

[B85] Staudacher E Distribution and morphology of descending brain neurons in the cricket *Gryllus bimaculatus* Cell and Tissue Research 1998 294 187 202 10.1007/s004410051169 9724469

[B86] Staudacher E Schildberger K A newly described neuropile in the deutocerebrum of the cricket: antennal afferents and descending interneurons Zoology 1999 102 212 226

[B87] Ignell R Dekker T Ghaninia M Hansson BS Neuronal architecture of the mosquito deutocerebrum The Journal of Comparative Neurology 2005 493 207 240 10.1002/cne.20800 16255032

[B88] Bräunig P Pflüger HJ Hustert R The specificity of central nervous projections of locust mechanoreceptors The Journal of Comparative Neurology 1983 218 197 207 10.1002/cne.902180207 6886072

[B89] Homberg U Rathmayer W Distribution of neurotransmitters in the insect brain 1994 40 Stuttgart: Gustav Fischer

[B90] Barrozo RB Couton L Lazzari CR Insausti TC Minoli SA Fresquet N Rospars JP Anton S Antennal pathways in the central nervous system of a blood-sucking bug, *Rhodnius prolixus* Arthropod Structure & Development 2009 38 101 110 10.1016/j.asd.2008.08.004 18809510

[B91] Damen WGM Hausdorf M Seyfarth EA Tautz D A conserved mode of head segmentation in arthropods revealed by the expression pattern of Hox genes in a spider Proceedings of the National Academy of Sciences 1998 95 10665 10670 10.1073/pnas.95.18.10665 PMC27952 9724761

[B92] Telford MJ Thomas RH Expression of homeobox genes shows chelicerate arthropods retain their deutocerebral segment Proceedings of the National Academy of Sciences 1998 95 10671 10675 10.1073/pnas.95.18.10671 PMC27953 9724762

[B93] Mittmann B Scholtz G Development of the nervous system in the "head" of *Limulus polyphemus *(Chelicerata: Xiphosura): morphological evidence for a correspondence between the segments of the chelicerae and of the (first) antennae of Mandibulata Development Genes and Evolution 2003 213 9 17 12590348 10.1007/s00427-002-0285-5

[B94] Eriksson BJ Budd GE Onychophoran cephalic nerves and their bearing on our understanding of head segmentation and stem-group evolution of Arthropoda Arthropod Structure and Development 2000 29 197 209 10.1016/S1467-8039(00)00027-X 18088927

[B95] Scholtz G Edgecombe GD Koenemann S, Jenner RA Heads, Hox and the phylogentic position of trilobites Crustacea and arthropod relationships 2005 Boca Raton: CRC Press 139 165

[B96] Scholz G Edgecombe GD The evolution of arthropod heads: reconciling morphological, developmental and palaeontological evidence Development Genes and Evolution 2006 216 395 415 10.1007/s00427-006-0085-4 16816969

[B97] Mayer G Koch M Ultrastructure and fate of the nephridial anlagen in the antennal segment of *Epiperipatus biolleyi *(Onychophora, Peripatidae) evidence for the onychophoran antennae being modified legs Arthropod Structure & Development 2005 34 471 480 10.1016/j.asd.2005.03.00422214827

[B98] Strausfeld NJ Strausfeld CM Loesel R Rowell D Stowe S Arthropod phylogeny: onychophoran brain organization suggests an archaic relationship with a chelicerate stem linage Proceedings of the Royal Society B 2006 273 1857 1966 10.1098/rspb.2006.3536 16822744 PMC1634797

[B99] Strausfeld NJ Strausfeld CM Stowe S Rowell D Loesel R The organization and evolutionary implications of neuropils and their neurons in the brain of the onychophoran *Euperipatoides rowelli* Arthropod Structure & Development 2006 35 169 196 10.1016/j.asd.2006.06.002 18089068

[B100] Harzsch S Glötzner J An immunohistochemical study on structure and development of the nervous system in the brine shrimp *Artemia salina *Linnaeus, 1758 (Branchiopoda, Anostraca) with remarks on the evolution of the arthropod brain Arthropod Structure & Development 2002 30 251 270 10.1016/S1467-8039(02)00012-9 18088960

[B101] Fritsch M Richter S The formation of the nervous system during larval development in *Triops cancriformis *(Bosc) (Crustacea, Branchiopoda): An immunohistochemical survey Journal of Morphology 2010 271 1457 1481 10.1002/jmor.10892 20938985

[B102] Stegner MEJ Richter S Morphology of the brain in *Hutchinsoniella macracantha *(Cephalocarida, Crustacea) Arthropod Structure & Development 2011 40 221 243 10.1016/j.asd.2011.04.001 21679884

